# Perioperative real-life mobility assessment using wearable step counts in idiopathic normal pressure hydrocephalus after ventriculoperitoneal shunt surgery: a prospective pilot study

**DOI:** 10.1007/s10143-026-04432-5

**Published:** 2026-08-13

**Authors:** Džiugas Meška, Jan Lost, Lisa Haddad, Hanibaal Dib, Kerim Beseoğlu, Thomas Beez

**Affiliations:** 1 Department of Neurosurgery, Medical Faculty, University Hospital Düsseldorf, Heinrich-Heine-University Düsseldorf, Düsseldorf, Germany; 2 Department of Neurosurgery and Spine Surgery, University Hospital Essen, University of Duisburg-Essen, Essen, Germany

**Keywords:** Idiopathic normal pressure hydrocephalus, Ventriculoperitoneal shunt, Wearable devices, Accelerometry, Mobility, Step count

## Abstract

Idiopathic normal pressure hydrocephalus (iNPH) is commonly treated with ventriculoperitoneal shunt (VPS) implantation, yet accurate patient selection remains challenging because conventional diagnostic tests are variably standardized and partly subjective. We evaluated whether wearable step-count monitoring may provide an objective assessment of real-life mobility and help identify patterns of postoperative mobility improvement. In this prospective pilot study, perioperative mobility was recorded using a wrist-worn wearable tracker (Garmin Vivofit 4). The primary endpoint was change in mean daily step count after VPS implantation. Secondary analyses included comparison between objective responders and non-responders, characterization of the postoperative time course of mobility change, association between objective and subjective clinical response, and exploratory receiver operating characteristic (ROC) analysis to identify a preoperative step-count threshold associated with postoperative mobility improvement. Clinical trial number: not applicable. Twelve patients completed the study protocol. In the overall cohort, postoperative step count increased numerically but did not differ significantly from baseline. Objective responders (*n* = 8) showed a significant increase in daily step count from 1656 ± 356 to 2311 ± 373 steps/day (*p* < 0.05), whereas non-responders (*n* = 4) showed no improvement. Responders had significantly lower preoperative daily step counts than non-responders (median 1118 [IQR 563–1869.5] vs. 5148 [IQR 4202.25–5796.5] steps/day; *p* = 0.049). In responders, mobility gains became significant from postoperative week 9 onward (F(12,84) = 9.75, *p* < 0.001), peaking at approximately 2.5-fold baseline by week 11. Objective mobility response was associated with subjective clinical improvement: 7 of 8 objective responders (87.5%) reported postoperative benefit, with a significant linear-by-linear association between objective and subjective outcome (*p* = 0.033). Exploratory ROC analysis identified a preoperative step-count threshold of 1881 steps/day (AUC = 0.875). Wearable step-count monitoring may enable objective assessment of real-life mobility in iNPH and could complement perioperative evaluation after VPS implantation. Larger prospective studies are needed to validate its role in patient selection and postoperative monitoring.

**Supplementary Information:**

The online version contains supplementary material available at 10.1007/s10143-026-04432-5.

## Introduction

Idiopathic normal pressure hydrocephalus (iNPH) is a potentially reversible neurological disorder of older adults characterized by the clinical triad of gait disturbance, cognitive decline, and urinary incontinence. Because these symptoms are highly prevalent in the elderly population and may also result from other neurological or systemic conditions, diagnosis and prognostication remain challenging [1–4]. Imaging findings such as ventriculomegaly and supportive markers including disproportionately enlarged subarachnoid spaces (DESH) may strengthen diagnostic suspicion, but these features are not specific and may also occur in other neurological disorders or in the setting of cerebral atrophy [5]. These uncertainties likely contribute to the wide variation in reported incidence, while the increasing number of shunt procedures performed in recent years underlines the clinical relevance of this disease [6, 7].

A broad range of clinical variables, biomarkers, neuroradiological signs, and advanced imaging techniques has been investigated to improve diagnostic precision in iNPH, but many of these approaches show limited sensitivity and specificity and are even less reliable in predicting individual treatment response [8–10]. In routine clinical practice, lumbar puncture with cerebrospinal fluid (CSF) drainage remains a key diagnostic tool for supporting the diagnosis and estimating the likelihood of benefit from CSF diversion [11]. However, even the CSF tap test is not uniformly standardized with regard to the amount of CSF removed, timing of reassessment, outcome measures, or response thresholds [12]. In addition, interpretation is partly based on brief clinical examinations and may be influenced by subjective perception and expectation of both examiner and patient. In contrast, daily step count may provide a simple and objective measure of real-life mobility and could capture functional change beyond short-term tap-test performance. The authors found sustained improvement in only 32% of patients after 36 months [13]. Moreover, in 25% of patients, the diagnosis was revised to dementias, such as Alzheimer’s disease, during the follow-up period. These findings suggest that currently used “classical” diagnostic criteria may be insufficient in a relevant subset of patients, and that patient-centered objective parameters could help address this gap.

Recent advancements in smart wearable devices have introduced continuous biometric monitoring into clinical practice, shifting the paradigm from subjective, discrete assessments to objective digital fingerprints of patient function [14]. In the broader neurological context, wearables facilitate support stroke rehabilitation and assessment of upper-limb motor impairment [15], monitor motor symptoms and pharmacodynamic responses in Parkinson’s disease [16], and enable automated home-based seizure detection in epilepsy [17]. Furthermore, accelerometry-based methodologies help characterize altered gait after incomplete spinal cord injury [18]. Neurosurgical spine applications have been explored utilizing daily step counts and gait velocity to track functional recovery after laminectomy and identify distinct preoperative functional decline phenotypes for decompression versus fusion indications [19, 20]. Real-time monitoring has been successfully employed to detect postoperative complications such as recurrent disc herniation, where an acute drop in objective activity metrics facilitated diagnosis before scheduled clinical follow-ups [21]. Furthermore, tri-axial sensors can classify spinal postures with 100% accuracy and monitor cervical curvature levels [22]. Beyond the spine, objective gait improvements have been quantified following carotid endarterectomy, and smartphone-based applications have validated tremor reduction after deep brain stimulation [23, 24]. Physiologic monitoring via wrist-mounted devices has also demonstrated high adherence and provided critical postoperative data after transsphenoidal surgery [25]. Although accelerometry and wearable sensors have been applied across several neurological and neurosurgical conditions, no prior study has specifically evaluated whether preoperative real-life daily step count can inform surgical suitability or expected postoperative benefit in patients with idiopathic normal pressure hydrocephalus.

In the present prospective pilot study, we evaluated perioperative real-life outpatient mobility in patients with iNPH using wearable accelerometric devices. We aimed to quantify preoperative and postoperative step counts, identify objective responder and non-responder patterns, characterize the postoperative time course of mobility change, and explore whether preoperative step count may help identify patients more likely to demonstrate postoperative mobility improvement.

## Materials and methods

### Study design and patient population

This prospective pilot study was conducted at a tertiary referral center between May 2018 and December 2022. Consecutive patients evaluated for suspected idiopathic normal pressure hydrocephalus (iNPH) were screened for eligibility. The study was approved by the institutional ethics committee of Heinrich Heine University Düsseldorf (registration number: 2018064730) and was performed in accordance with the Declaration of Helsinki and its later amendments. Written informed consent was obtained from all participants or their legally authorized representatives prior to study enrolment.

**Fig. 1:**
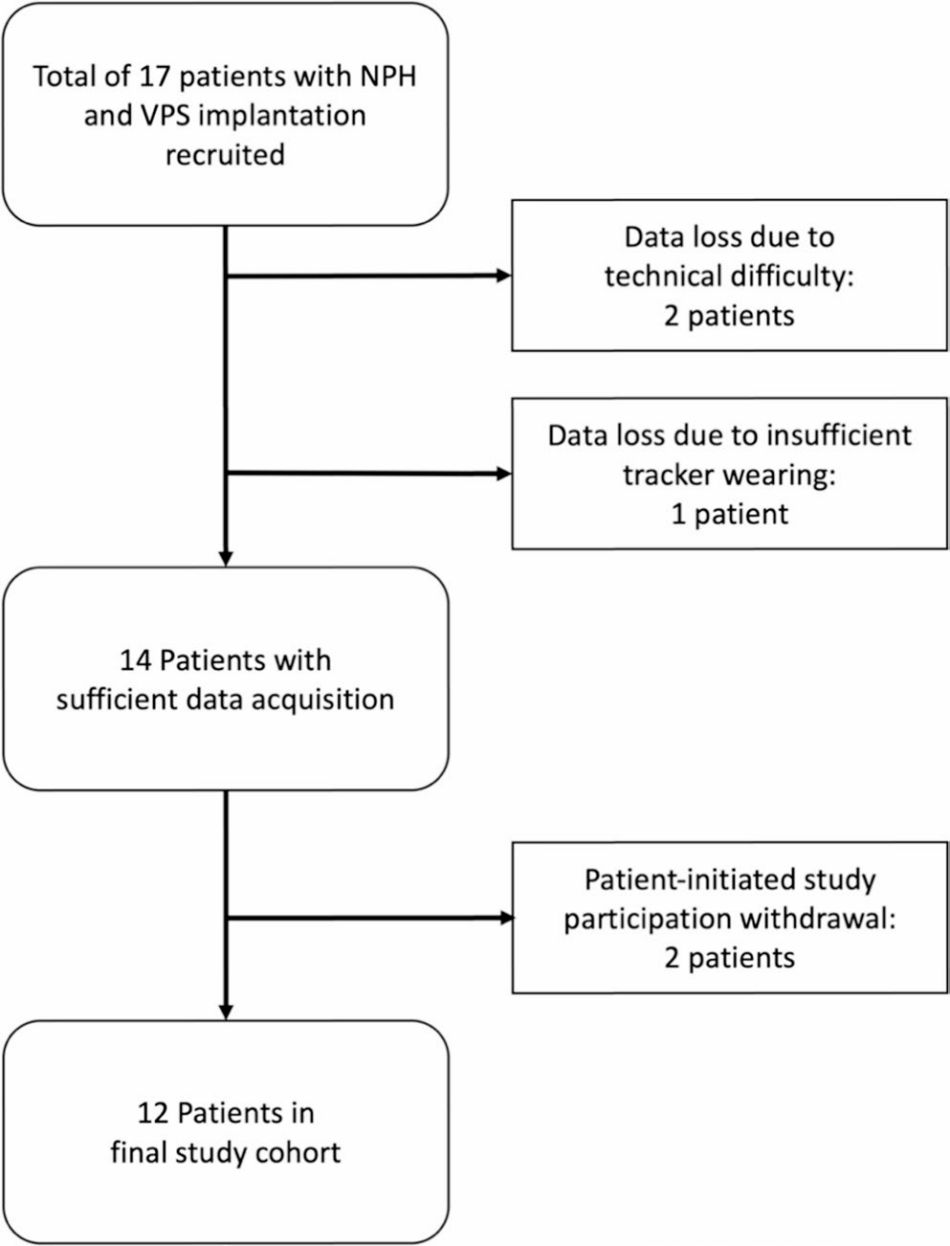
Flowchart of patient selection and study inclusion

Seventeen patients were initially included. Two patients discontinued participation early because of technical problems, two withdrew because the wearable device interfered with their daily activities, and one patient was excluded because the device was not worn preoperatively, resulting in missing baseline data. Twelve patients completed the study protocol and were included in the final analysis. The patient selection process is shown in Fig. 1.

### Patient selection

Consecutive patients evaluated for suspected iNPH at University Hospital Düsseldorf were screened for inclusion. Inclusion criteria were: (1) clinical suspicion of iNPH with complete or incomplete Hakim’s triad and at least one supportive imaging feature, including ventriculomegaly, disproportionately enlarged subarachnoid spaces (DESH), or a callosal angle of 50°–80°; (2) a positive cerebrospinal fluid (CSF) tap test according to the institutional protocol, defined by removal of 30 mL CSF followed by improvement in gait on the 10-m stand-up-and-go test and/or improvement in Mini-Mental State Examination (MMSE) score within 24 h; (3) a clinical decision to proceed with ventriculoperitoneal shunt (VPS) implantation; (4) written informed consent; and (5) sufficient adherence to perioperative wearable monitoring to allow analysis of preoperative and postoperative step-count data.

Exclusion criteria were refusal or withdrawal of consent, technical failure preventing data acquisition, intolerance of the wearable device, and noncompliance with device wear resulting in substantial missing data. Only patients who completed the protocol with adequate accelerometry datasets were included in the final analysis.

### Diagnostic work-up, surgery, and monitoring protocol

**Fig. 2:**
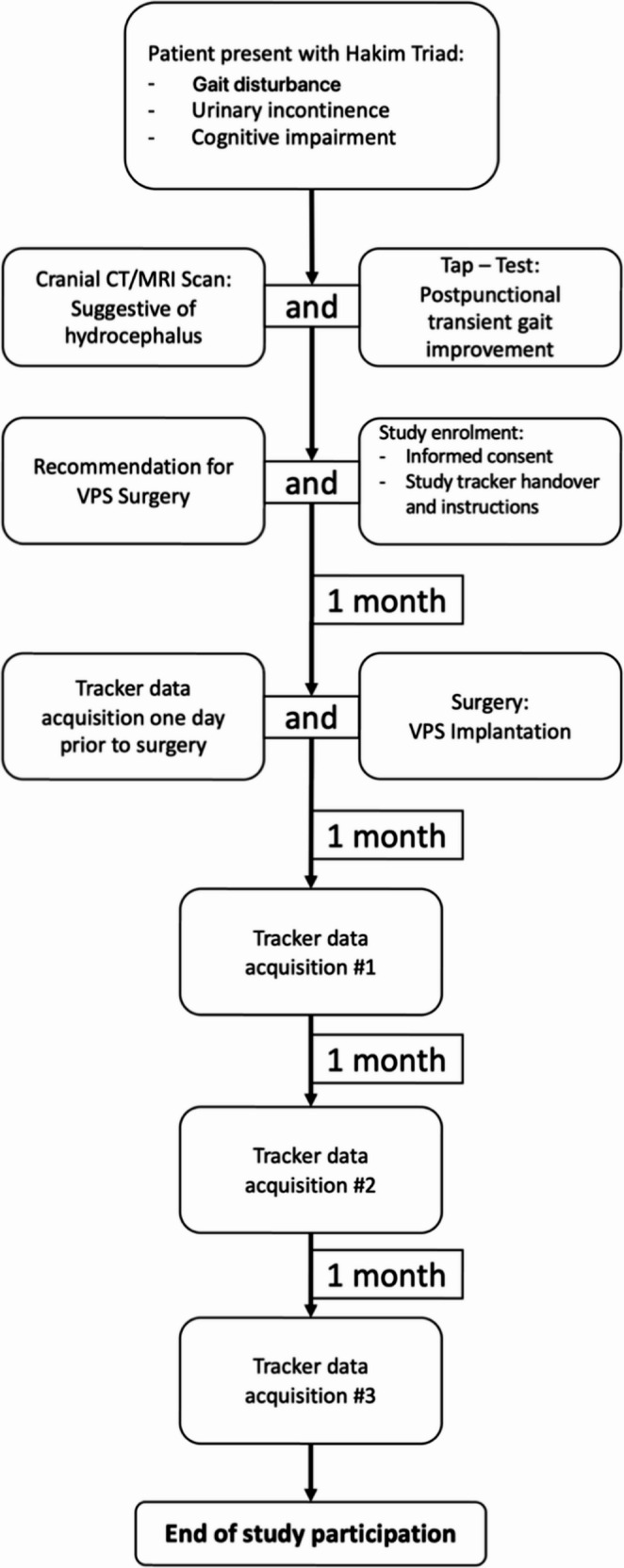
Flowchart of diagnostic work-up, treatment course, and wearable monitoring protocol in patients with idiopathic normal pressure hydrocephalus

Patients with suspected iNPH underwent standardized diagnostic work-up, including a CSF tap test, in accordance with the institutional protocol and international guidelines (Fig. 2). A positive response to the tap test was defined as any improvement in gait performance on the 10-m stand-up-and-go test, reflected by a reduction in time and/or number of steps required to complete the test, and/or improvement in cognitive performance on MMSE within 24 h after drainage of 30 mL CSF.

After confirmation of iNPH and consent to VPS surgery, patients were enrolled in the study and provided with a wrist-worn accelerometry tracker (Garmin Vivofit 4, Garmin Ltd., USA) one month before surgery for continuous recording of daily step count. The device records tri-axial acceleration signals, which are processed by the manufacturer’s proprietary algorithms to detect step events and aggregate them into daily step counts. Baseline mobility data were downloaded one day before surgery.

VPS implantation was performed according to institutional standard and included ultrasound-guided ventricular catheter placement, use of antibiotic-impregnated catheters (Codman Bactiseal, Integra LifeSciences, USA), and implantation of adjustable gravitational valves (proGAV 2.0, Christoph Miethke, Germany). Postoperative computed tomography was performed to verify catheter position. Wearable data were downloaded monthly for 3 months after surgery. After the final download, patients rated their overall clinical change as better, unchanged, or worse compared with their preoperative status. Study participation then ended, and further follow-up continued as part of routine clinical care.

### Data collection

Clinical, epidemiological, and imaging data were collected prospectively and supplemented from the electronic health record where necessary. Recorded clinical variables included age, sex, symptom duration, components of Hakim’s triad (gait disturbance, cognitive impairment, and urinary incontinence), body mass index, comorbidity burden as assessed by the Charlson Comorbidity Index, and perioperative risk classification according to the American Society of Anesthesiologists (ASA) score. Radiological variables included the Evans index, presence of DESH, and the callosal angle measured on preoperative neuroimaging. Clinical outcome measures included gait performance during the tap test, MMSE score, and postoperative patient-reported global clinical response.

Accelerometry data were expressed as daily step count. Data obtained one day before surgery covered the preceding four weeks and were used as the preoperative baseline period. Postoperative data were downloaded monthly for three months. Because the device continuously recorded daily step counts, postoperative mobility could additionally be aggregated into weekly intervals for longitudinal analysis.

### Outcomes

The primary endpoint was the change in mean daily step count after VPS implantation. Secondary analyses included: (1) subgroup comparison between objective responders and non-responders; (2) characterization of the postoperative time course of mobility change in responders; (3) association between objective mobility response and subjective clinical response; and (4) exploratory receiver operating characteristic (ROC) analysis to identify a preoperative step-count threshold associated with postoperative mobility improvement.

Objective response was defined a priori as a sustained postoperative increase in mean daily step count compared with the individual preoperative baseline across the 3-month postoperative monitoring period. Patients were classified as objective responders only if their mean postoperative daily step count over the entire follow-up period exceeded their preoperative baseline and the longitudinal mobility trajectory showed a persistent upward shift rather than an isolated transient fluctuation. Patients with unchanged or declining postoperative mobility were classified as non-responders. The preoperative-to-postoperative change within the responder subgroup was subsequently evaluated using a paired t-test.

### Statistical analysis

For each patient, mean daily step count was calculated for the preoperative and postoperative periods and expressed as absolute values (steps/day). For descriptive visualization, postoperative mobility was additionally expressed as standardized step count, defined as the percentage of the individual patient’s preoperative mean daily step count. Unless otherwise indicated, continuous variables are reported as mean ± standard error of the mean or median (interquartile range), as appropriate.

Preoperative and postoperative mean daily step counts were compared using paired t-tests. Weekly postoperative step counts were aggregated into 13 repeated measurements and analyzed using repeated-measures analysis of variance (ANOVA). Sphericity was assessed, and Bonferroni correction was applied where appropriate for post hoc comparisons.

Exploratory Spearman correlation analysis was performed to examine potential associations between preoperative clinical or radiological variables and subjective clinical response (Online Resource 1). Given the small sample size, these analyses were interpreted descriptively. Associations between objective accelerometry-based mobility response and subjective clinical outcome were examined using the chi-square test of independence. For ordered categories, the linear-by-linear association test was additionally applied. Effect size for contingency tables was expressed as Cramer’s V, with values of 0.1, 0.3, and 0.5 considered small, moderate, and strong, respectively.

Exploratory comparisons of baseline clinical, radiological, and functional variables between objective responders and non-responders were performed using the Mann–Whitney U test for continuous variables and Fisher’s exact test for categorical variables.

To explore the discriminatory performance of preoperative step count for objective postoperative mobility response, ROC analysis was performed with responder status defined by accelerometry-based mobility response as the positive state. The optimal cut-off was determined using Youden’s index (J = sensitivity + specificity − 1). Area under the curve (AUC), sensitivity, and specificity were reported. All analyses were performed using SPSS version 27 (IBM Corp., Armonk, NY, USA), and statistical significance was set at α = 0.05.

### Reporting guideline

Reporting of this observational study followed the STROBE (Strengthening the Reporting of Observational Studies in Epidemiology) guideline [26].

## Results

### Patient demographics and baseline clinical characteristics

The final cohort comprised 12 patients. Median age at surgery was 70 years (interquartile range [IQR] 63.5–76.0), and 4 patients (33.3%) were female. All patients presented with gait impairment, whereas urinary incontinence and cognitive decline were present in 10 patients (83.3%) and 11 patients (91.7%), respectively. A complete Hakim triad was observed in 10 patients (83.3%).

Median symptom duration before surgery was 1.25 years (IQR 1.0–2.0). Median body mass index was 26.5 (IQR 24.0–29.0), and clinically relevant comorbidity, defined as an American Society of Anesthesiologists score > 2 and/or Charlson Comorbidity Index ≥ 4, was present in 6 patients (50.0%).

Preoperative neuroimaging showed a median Evans index of 0.395 (IQR 0.36–0.44) and a median callosal angle of 64.05° (IQR 52.1–71.1). Disproportionately enlarged subarachnoid spaces were present in 6 patients (50.0%). After ventriculoperitoneal shunt implantation, 8 patients (66.7%) reported subjective clinical improvement, 3 patients (25.0%) remained unchanged, and 1 patient (8.3%) reported clinical deterioration.

### Mobility before versus after surgery in the overall cohort

Mean daily step count increased numerically after shunt implantation but did not differ significantly from baseline. Preoperative mean daily step count was 2349 ± 466 steps/day, compared with 2595 ± 406 steps/day postoperatively (paired t-test: t(11) = − 0.984, *p* = 0.346).

### Responders versus non-responders based on mobility assessment

**Fig. 3:**
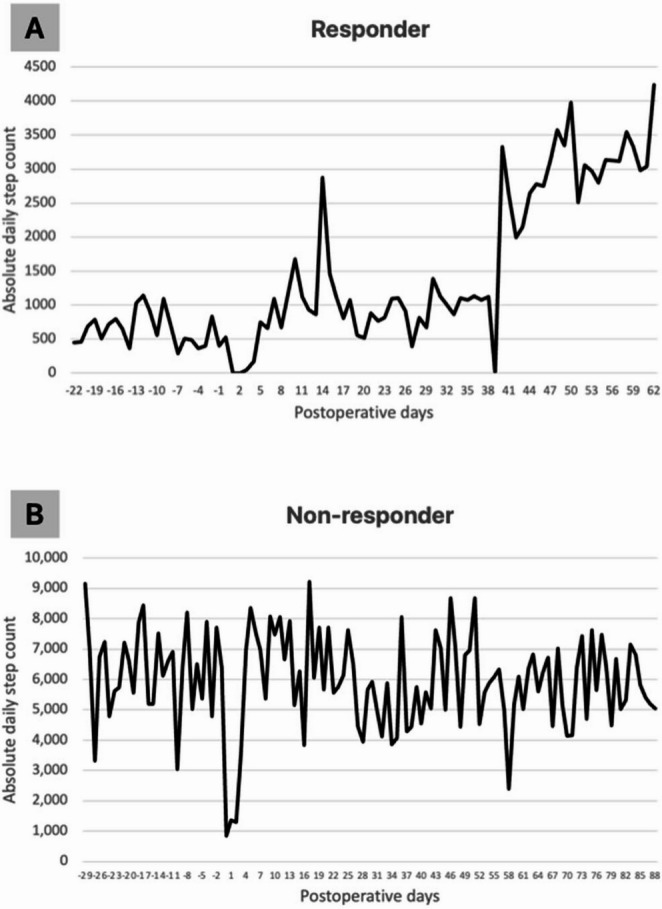
Examples of postoperative mobility trajectories. (**A**) Objective responder with a clear postoperative increase in daily step count. (**B**) Non-responder without postoperative mobility improvement

**Fig. 4:**
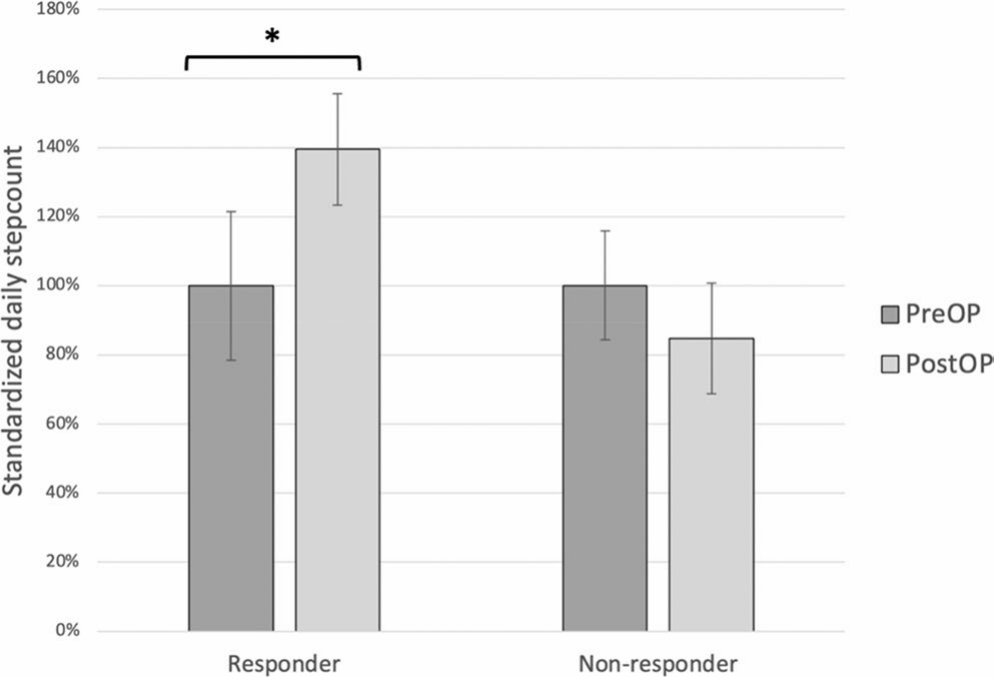
Preoperative and postoperative mean daily step count in objective responders and non-responders. In responders, postoperative mobility increased to approximately 140% of baseline

Mobility trajectories revealed two subgroups: objective responders and non-responders (Fig. 3). Objective responders showed a significant increase in postoperative daily step count compared with baseline (2311 ± 373 vs. 1656 ± 356 steps/day; paired t-test: t(7) = 2.542, *p* < 0.05), corresponding to an average increase to approximately 140% of baseline mobility across the entire 3-month postoperative follow-up period (Fig. 4). In contrast, non-responders showed no postoperative improvement.

### Exploratory comparison of baseline characteristics between objective responders and non-responders

To further evaluate whether objective responders and non-responders differed in established preoperative clinical or radiological characteristics, exploratory between-group comparisons were performed. Objective responders had significantly lower preoperative daily step counts than non-responders (median 1118 [IQR 563–1869.5] vs. 5148 [IQR 4202.25–5796.5] steps/day; Mann–Whitney U = 4.0, *p* = 0.049). In contrast, no significant differences were observed for age, symptom duration, BMI, Charlson Comorbidity Index, ASA score, Evans index, callosal angle, complete Hakim triad, urinary incontinence, cognitive impairment, or DESH. Because of the small sample size, these analyses were interpreted descriptively and should be considered hypothesis-generating. Detailed exploratory comparisons of continuous and categorical baseline variables are provided in Online Resources 2 and 3.

### Time course of mobility improvement in responders

**Fig. 5:**
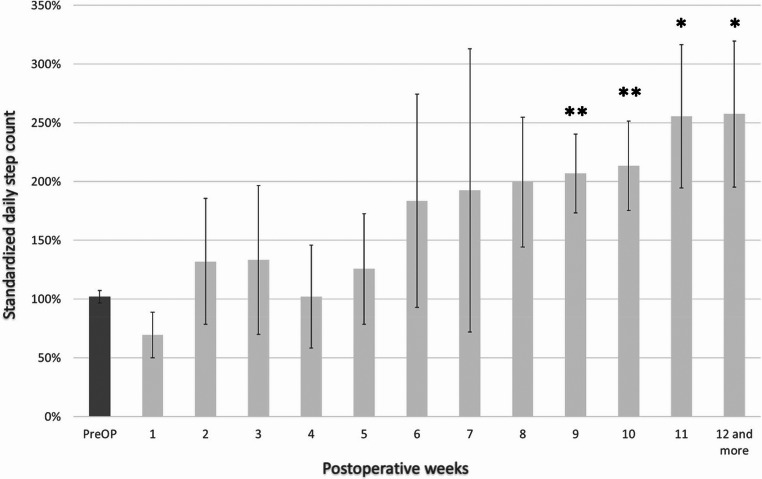
Time course of postoperative mobility change in objective responders. Significant increases in daily step count emerged from postoperative week 9 onward, with a peak at approximately 2.5-fold baseline around week 11

To characterize the temporal pattern of postoperative recovery, the follow-up period in responders was analyzed in weekly intervals. Repeated-measures analysis of variance demonstrated a significant change in daily step count across follow-up (F(12,84) = 9.747, *p* < 0.001) (Fig. 5). Post hoc comparisons showed significant differences between preoperative baseline and postoperative week 9 (D = 1.049, *p* = 0.008), week 10 (D = 1.113, *p* = 0.007), week 11 (D = 1.535, *p* = 0.019), and week ≥ 12 (D = 1.555, *p* = 0.026). In the weekly trajectory analysis, daily step count reached a peak of approximately 2.5-fold baseline around postoperative week 11 before stabilizing.

### Association between objective mobility response and subjective clinical response

**Fig. 6:**
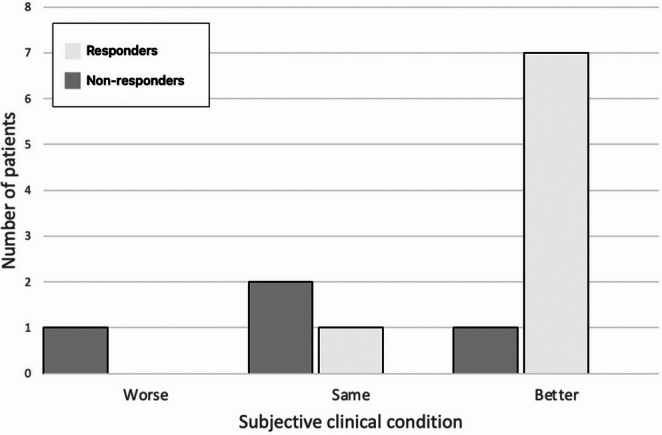
Association between objective mobility response and subjective clinical outcome. Most objective responders reported subjective clinical improvement

Objective mobility response was associated with patient-reported subjective outcome (Fig. 6). The overall association between objective responder status and subjective clinical response approached statistical significance (χ²(2, *N* = 12) = 5.06, *p* = 0.080), with a strong effect size (Cramer’s V = 0.65). In addition, a significant linear-by-linear association was observed (χ²(1) = 4.57, *p* = 0.033), indicating that subjective improvement increased in parallel with objective mobility gains. Among objective responders, 7 of 8 patients (87.5%) reported subjective clinical improvement.

### Predicting postoperative mobility improvement

**Fig. 7:**
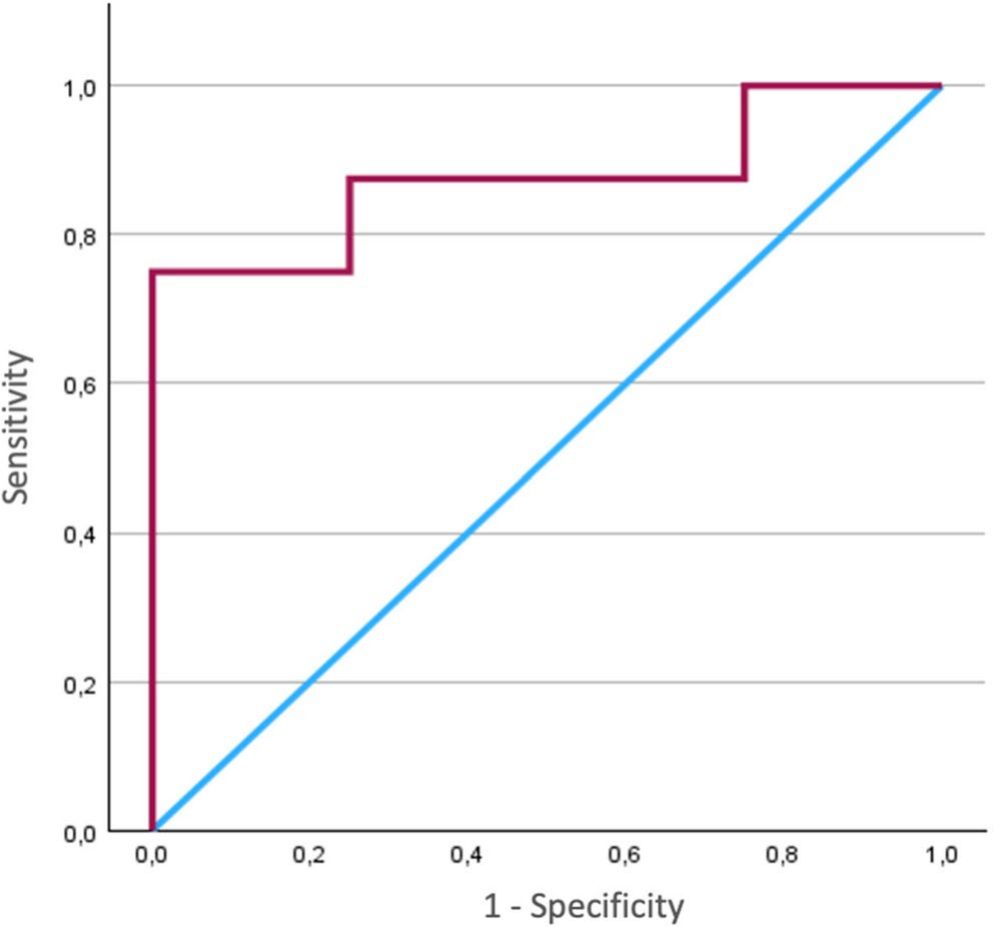
Exploratory receiver operating characteristic curve illustrating the discriminative ability of preoperative daily step count to identify postoperative objective responders. The area under the curve was 0.875

Exploratory receiver operating characteristic analysis identified a preoperative step-count threshold of 1881 steps/day for discrimination between objective mobility responders and non-responders within this cohort (Fig. 7). The area under the curve was 0.875, and the Youden-derived cut-off corresponded to 75% sensitivity and 100% specificity. Given the small sample size, this threshold should be interpreted as hypothesis-generating rather than as a clinically validated decision threshold.

## Discussion

This prospective pilot study evaluated daily step count as an objective surrogate of real-life mobility in patients undergoing VPS implantation for iNPH using a wearable device. Although the overall cohort showed only a numerical postoperative increase in mean daily step count, individual mobility trajectories suggested a subgroup of objective responders who demonstrated increased postoperative mobility, with postoperative daily step count increasing to approximately 140% of the individual preoperative baseline. In these responders, mobility gains became statistically significant from postoperative week 9 onward, with maximal improvement observed around week 11, when daily step counts reached the maximum of 2.5-fold baseline before stabilizing. Objective mobility response was directionally aligned with subjective clinical improvement, with a strong effect size and a significant linear-by-linear association. Exploratory ROC analysis further suggested that lower preoperative step counts may be associated with a higher likelihood of postoperative mobility improvement.

Compared with conventional diagnostic approaches in iNPH, wearable step-count monitoring offers an objective and patient-centered assessment of mobility under real-life conditions. A growing body of literature has reported the feasibility of personal digital devices in outpatient neurological monitoring [27]. In iNPH, prior work has assessed the accuracy of commercial activity monitors in counting steps [28], whereas other studies have used dedicated sensor-based systems to characterize gait in controlled laboratory environments [29, 30]. By contrast, the present study focused on longitudinal perioperative mobility trajectories in everyday life using a simple commercially available wearable device. Further studies describe the use of such devices to record everyday mobility in patients with ischemic stroke, Parkinson’s disease and multiple sclerosis [31–34].

Real-life outpatient mobility before and after surgery has only rarely been assessed using such devices. Although a minority of initially recruited patients discontinued participation because of technical problems, discomfort, or noncompliance, the feasibility of wearable monitoring appeared acceptable among patients who completed the protocol. In addition, such devices are widely available, relatively inexpensive, and easy to integrate into clinical follow-up without the need for highly specialized infrastructure.

Assessment under real-life conditions may represent a relevant advantage over laboratory-based mobility evaluation. Renggli et al. demonstrated that gait characteristics in older adults differ between controlled laboratory settings and real-world environments, with real-life assessment better reflecting functionally relevant differences [35]. This supports the concept that mobility in natural surroundings may provide clinically meaningful information beyond standardized short-term examinations.

The results of the present pilot study suggest that simple wearable trackers may help identify patients with profound reduction of mobility, within a cohort of patients fulfilling the established diagnostic criteria of iNPH. In this pilot cohort, this subset of patients appeared more likely to experience objective mobility improvement after shunt surgery; 88% of responders based on step count also reported subjective clinical benefit. Additionally, the study provides insight into the extent of mobility improvement and its time course after VPS surgery. Alternatively, these patients might have been misdiagnosed and thus did not respond to VPS implantation – a hypothesis that certainly requires further investigation, but was raised as well by other authors [13]. In addition, future studies should evaluate whether objective mobility data could be incorporated into the preoperative diagnostic work-up of iNPH, for example based on daily step count as a differentiation from other neurodegenerative conditions. In the future, the knowledge gained from this study might also be used to optimize VPS therapy in further aspects apart from patient selection. For example, adjusting the shunt valve opening pressure according to objective data of daily patient mobility at home could have advantages over the status quo of guiding valve adjustments according to subjective description of over- or underdrainage symptoms by the patient or simply based on expert opinion and experience.

The preoperative step-count threshold identified in this study should be interpreted with caution. Although the cut-off of 1881 steps/day showed good discriminatory performance within this cohort, it was derived from a small single-center pilot sample and has not been externally validated. It should therefore be regarded as exploratory and hypothesis-generating rather than as a clinically applicable decision threshold. Future studies should examine whether wearable-derived mobility measures retain predictive value in larger and independent cohorts and how they relate to established clinical, radiological, and CSF-based assessments.

Beyond patient selection, objective longitudinal mobility monitoring may also have potential for postoperative follow-up. In principle, home-based mobility trends could complement symptom-based assessment and might eventually contribute to more data-driven shunt valve management. However, whether wearable step-count trajectories can reliably guide such decisions requires prospective validation.

### Limitations

This study has several limitations. First, it was a small single-center pilot study, which limits statistical power, precision, and generalizability and increases susceptibility to random variation. Second, the classification of objective response was based on postoperative change in step count over a three-month follow-up period and may have been influenced by factors unrelated to iNPH itself, including intercurrent illness, seasonal effects, behavioral adaptation, or differences in daily routines. Third, outcome assessment was not blinded, and subjective clinical response was based on patient-reported postoperative evaluation rather than blinded adjudication. Fourth, no standardized rehabilitation or physical activity control protocol was implemented, so postoperative mobility trajectories may have been influenced by differences in recovery behavior or supportive care. Fifth, the wearable device relies on proprietary step-detection algorithms that cannot be independently audited, and step count alone does not capture gait quality, stride length, or balance. Sixth, exact daily wearing time was not available; therefore, a residual influence of differences in device wear time on recorded step counts cannot be completely excluded. Finally, the preoperative ROC-derived threshold may overfit this cohort and requires external validation in larger prospective studies.

## Conclusion

Wearable step-count monitoring enables objective assessment of real-life mobility in patients with iNPH before and after VPS implantation. In this pilot cohort, an objective responder subgroup showed significant mobility gains emerging from postoperative week 9 and peaking around week 11. An exploratory preoperative threshold of 1881 steps/day showed good discriminatory performance within this cohort, but requires external validation. Larger prospective studies are warranted to determine how wearable mobility metrics can be integrated into diagnostic evaluation, patient counseling, and postoperative follow-up in iNPH.

## Supplementary Information

Below is the link to the electronic supplementary material.

Supplementary File 1 (DOCX 146 KB)

## Data Availability

Anonymized individual participant data underlying the results of this study, including preoperative and postoperative accelerometry-derived mobility metrics, will be made available upon reasonable request to qualified investigators. Investigators requesting access must submit a methodologically sound proposal outlining the intended analyses. Following approval by the corresponding author, data will be shared via secure transfer under a data-sharing agreement. No identifying information will be released.
