# Supplementary material for: Perioperative real-life mobility assessment using wearable step counts in idiopathic normal pressure hydrocephalus after ventriculoperitoneal shunt surgery: a prospective pilot study
Source: Neurosurg Rev. 2026 Aug 13;49(1):521. doi: 10.1007/s10143-026-04432-5 (PMC13469384; doi:10.1007/s10143-026-04432-5)
Supplement: Supplementary file 1 [file 10143_2026_4432_MOESM1_ESM.pdf]

Online Resources

Online Resource 1. Exploratory Spearman correlation analysis of variables associated with patient-reported subjective clinical response.

			**Subjective Clinical Response**
**Spearman's rho**	**Preoperative daily step count**	Correlation Coefficient	-.290
		Sig. (2-tailed)	.361
		N	12
	**Objective responder status**	Correlation Coefficient	.646*
		Sig. (2-tailed)	**.023**
		N	12
	**Subjective Clinical Response**	Correlation Coefficient	1.000
		Sig. (2-tailed)	
		N	12
	**Age**	Correlation Coefficient	.186
		Sig. (2-tailed)	.564
		N	12
	**Hakim Triad**	Correlation Coefficient	.272
		Sig. (2-tailed)	.392
		N	12
	**Incontinence**	Correlation Coefficient	.272
		Sig. (2-tailed)	.392
		N	12
	**Cognitive Deficit**	Correlation Coefficient	.577*
		Sig. (2-tailed)	.049
		N	12
	**Symptom Duration in Years**	Correlation Coefficient	-.218
		Sig. (2-tailed)	.495
		N	12
	**BMI**	Correlation Coefficient	.346
		Sig. (2-tailed)	.270
		N	12
	**Charlson Comorbidity Index**	Correlation Coefficient	.086
		Sig. (2-tailed)	.791
		N	12
	**ASA Score**	Correlation Coefficient	.055
		Sig. (2-tailed)	.865
		N	12
	**Evans Index**	Correlation Coefficient	-.053
		Sig. (2-tailed)	.871
		N	12
	**DESH**	Correlation Coefficient	-.261
		Sig. (2-tailed)	.412
		N	12

	**Callosal Angle**	Correlation Coefficient	.214
		Sig. (2-tailed)	.504
		N	12

Note: Two-tailed Spearman correlation coefficients (ρ) are reported. Analyses were exploratory and interpreted descriptively because of the small sample size.

Online Resource 2. Exploratory comparison of continuous baseline characteristics between objective responders and non-responders.

Variable	Objective responders n = 8, median (IQR)	Non-responders n = 4, median (IQR)	Mann–Whitney U	p-value
Preoperative daily step count, steps/day	1118 (563–1869.5)	5148 (4202.25–5796.5)	4.0	0.049
Age, years	73.5 (68.25–76.75)	64.0 (57.5–69.75)	25.5	0.125
Symptom duration, years	1.0 (1.0–2.0)	1.5 (1.38–1.88)	12.5	0.585
BMI	26.5 (25.75–28.0)	26.5 (23.75–29.75)	18.5	0.733
Charlson Comorbidity Index	3.5 (3.0–6.75)	1.5 (1.0–3.25)	26.0	0.099
ASA score	2.0 (2.0–3.0)	2.0 (1.75–2.25)	20.5	0.439
Evans index	0.38 (0.35–0.41)	0.43 (0.40–0.47)	6.0	0.105
Callosal angle, degrees	65.45 (59.9–68.85)	57.55 (50.75–67.83)	21.0	0.461

Note: Values are shown as median (interquartile range). Continuous variables were compared using the Mann–Whitney U test. Analyses were exploratory and interpreted descriptively because of the small sample size.

Online Resource 3. Exploratory comparison of categorical baseline characteristics between objective responders and non-responders.

Variable	Objective responders n = 8	Non-responders n = 4	p-value
Complete Hakim triad	7/8 (87.5%)	3/4 (75.0%)	1.000
Urinary incontinence	7/8 (87.5%)	3/4 (75.0%)	1.000

Variable	Objective responders n = 8	Non-responders n = 4	p-value
Cognitive impairment	8/8 (100.0%)	3/4 (75.0%)	0.333
DESH	4/8 (50.0%)	2/4 (50.0%)	1.000

Note: Values are shown as n (%). Categorical variables were compared using Fisher's exact test. Analyses were exploratory and interpreted descriptively because of the small sample size.
